# Reclassification of *Theileria annae* as *Babesia vulpes* sp. nov.

**DOI:** 10.1186/s13071-015-0830-5

**Published:** 2015-04-08

**Authors:** Gad Baneth, Monica Florin-Christensen, Luís Cardoso, Leonhard Schnittger

**Affiliations:** Koret School of Veterinary Medicine, Hebrew University, P.O. Box 12, Rehovot, 76100 Israel; Institute of Pathobiology, Center of Research in Veterinary and Agronomic Sciences, INTA-Castelar, 1686 Hurlingham, Argentina; CONICET, Ciudad Autónoma de Buenos Aires, Argentina; Department of Veterinary Sciences, School of Agrarian and Veterinary Sciences, University of Trás-os-Montes e Alto Douro (UTAD), Vila Real, Portugal

**Keywords:** *Babesia vulpes*, *Babesia microti*, *Babesia* cf. *microti*, *Babesia microti*-like, *Theileria annae*, *Babesia* (*Theileria*) *annae*, *Babesia annae*, *Babesia* Spanish dog isolate, red fox, dog

## Abstract

**Background:**

*Theileria annae* is a tick-transmitted small piroplasmid that infects dogs and foxes in North America and Europe. Due to disagreement on its placement in the *Theileria* or *Babesia* genera, several synonyms have been used for this parasite, including *Babesia* Spanish dog isolate, *Babesia microti*-like, *Babesia* (*Theileria*) *annae*, and *Babesia* cf. *microti*. Infections by this parasite cause anemia, thrombocytopenia, and azotemia in dogs but are mostly subclinical in red foxes (*Vulpes vulpes*). Furthermore, high infection rates have been detected among red fox populations in distant regions strongly suggesting that these canines act as the parasite’s natural host. This study aims to reassess and harmonize the phylogenetic placement and binomen of *T. annae* within the order Piroplasmida.

**Methods:**

Four molecular phylogenetic trees were constructed using a maximum likelihood algorithm based on DNA alignments of: (i) near-complete *18S rRNA* gene sequences (n = 76 and n = 93), (ii) near-complete and incomplete *18S rRNA* gene sequences (n = 92), and (iii) *tubulin-beta* gene sequences (n = 32) from *B. microti* and *B. microti*-related parasites including those detected in dogs and foxes.

**Results:**

All phylogenetic trees demonstrate that *T. annae* and its synonyms are not *Theileria* parasites but are most closely related with *B. microti*. The phylogenetic tree based on the *18S rRNA* gene forms two separate branches with high bootstrap value, of which one branch corresponds to *Babesia* species infecting rodents, humans, and macaques, while the other corresponds to species exclusively infecting carnivores. Within the carnivore group, *T. annae* and its synonyms from distant regions segregate into a single clade with a highly significant bootstrap value corroborating their separate species identity.

**Conclusion:**

Phylogenetic analysis clearly shows that *T. annae* and its synonyms do not pertain to *Theileria* and can be clearly defined as a separate species. Based on the facts that *T. annae* and its synonyms have not been shown to have a leukocyte stage, as expected in *Theileria*, do not infect humans and rodents as *B. microti*, and cluster phylogenetically as a separate species, this study proposes to name this parasite *Babesia vulpes* sp. nov., after its natural host, the red fox *V. vulpes*.

**Electronic supplementary material:**

The online version of this article (doi:10.1186/s13071-015-0830-5) contains supplementary material, which is available to authorized users.

## Background

*Babesia* and *Theileria* are tick-borne intracellular parasites that infect a variety of vertebrate hosts. Both, *Babesia* and *Theileria*, belong to the phylum Apicomplexa, class Piroplasmea, and order Piroplasmida. Despite their morphological resemblances and their similar intraerythrocytic life stage in the vertebrate host, they differ by a main characteristic feature of a pre-erythrocytic life stage in leukocytes found in *Theileria* but not in *Babesia*. Several species of piroplasmids infect domestic dogs and wild canines [1]. A relatively recent addition to the species of these genera was a small piroplasmid initially reported in a sick dog from Spain and shown to be most closely related with *Babesia microti* by phylogenetic analysis, for which it was first referred to as *Babesia microti*-like species [2]. Based on the observation that this pathogen did not segregate with *Babesia* parasites belonging to the *Babesia* sensu stricto group (reviewed in Schnittger et al. [3]), Zahler et al. [2] concluded that it belongs to the genus *Theileria* and proposed it to be named *Theileria annae* after the name of the corresponding author’s dog [2,4]. Shortly after its description, this pathogen was shown to cause severe disease with anemia, thrombocytopenia, and azotemia in 157 dogs from the Galicia region in northwestern Spain [5] and has since then been identified as a cause of infection and/or disease in dogs in other areas of northern Spain, Portugal, Croatia, Sweden, and USA [6-10].

Infection of red foxes (*Vulpes vulpes*) by *T. annae* was first recorded in Spain in 2003 [11] and subsequently in Italy, Croatia, Canada, USA, Portugal, Germany, and Austria [12-18]. Additionally, grey foxes (*Urocyon cinereoargenteus*) from the USA were also reported to be infected with *T. annae* [15]. The prevalence of infection found in red fox populations was often high with positive detection in 39% (50/127), 46% (121/261), 50% (18/26), and 69% (63/91) of foxes sampled in North America, Germany, Austria, and Portugal, respectively [15-18]. This is in contrast to the sporadic nature of domestic dog infection where only low prevalence rates comprising of single dogs were found in population surveys [7,10]. The observed high prevalence of *T. annae* infections of red foxes from different regions, with one exception from northern Italy [19], as well as the fact that they do not seem to cause severe disease in these animals have prompted scientists to suggest that red foxes are the natural reservoirs of this pathogen and a source for domestic dog infection [16-18,20]. DNA sequences of *T. annae* from foxes published to date or deposited in GenBank and as yet unpublished, are included in Additional file 1: Table S1.

The modes of transmission and tick vectors of *T. annae* have not been determined. It has been suggested that the hedgehog tick *Ixodes hexagonus* is a vector of this parasite, based on a survey of tick infestation of infected and uninfected dogs in northwestern Spain [21]. However, no transmission studies to corroborate this suspicion have been published. Furthermore, *T. annae* infection has been detected in areas where this tick species has not been reported [15]. *T. annae* DNA has been detected in several tick species including *I. hexagonus*, *I. ricinus* [17,22], *I. canisuga* [17], and *Rhipicephalus sanguineus* [23]. However, these findings do not provide positive proof for the capacity of these ticks to act as competent vectors for the parasite. On the other hand, they might suggest that the parasite can be transmitted by different tick species, as has been observed for other piroplasmids [3,24]. It has been suggested that other non-vectorial modes of natural transmission described for canine *Babesia* species, including transplacental transmission and direct infection by dog bites, as in the case of *Babesia gibsoni* [1,25], can also be valid for *T. annae* [15].

Since there is disagreement on the placement of *T. annae* within the *Theileria* genus [26], it has been named in different ways in a variety of publications. Synonyms of *T. annae* include *Babesia* Spanish dog isolate [10], *Babesia-microti*-like [2,15], *Babesia annae* [27,28], *Babesia* (*Theileria*) *annae* [14], and *Babesia* cf. *microti* [29]. In order to avoid ongoing confusion associated with the use of multiple names, the aim of this study was to: (i) reevaluate the placement of *T. annae* in the order Piroplasmida, and to (ii) rename it as to reflect its independent species status.

## Methods

A BLASTn search was carried out to identify and subsequently download all *B. microti* and *B. microti*-related *18S rRNA* gene sequences available in Genbank. Over 130 sequences were identified from which those that showed indications of sequence mistakes, or found to be too short to result in a relevant phylogenetic signal as judged by low bootstrap values during a preliminary analysis, were discarded. The remaining sequences were subjected to alignment using MUSCLE [30]. The analysis involved 76 *18S rRNA* nucleotide sequences of which all positions containing gaps and missing data were eliminated resulting in a total of 1,522 positions in the final dataset. After estimation of the shape parameter [31], the T92 + G + I model was applied to infer the tree. Phylogenetic analysis was carried out using the MEGA6 software [32].

## Results

**Figure 1:**
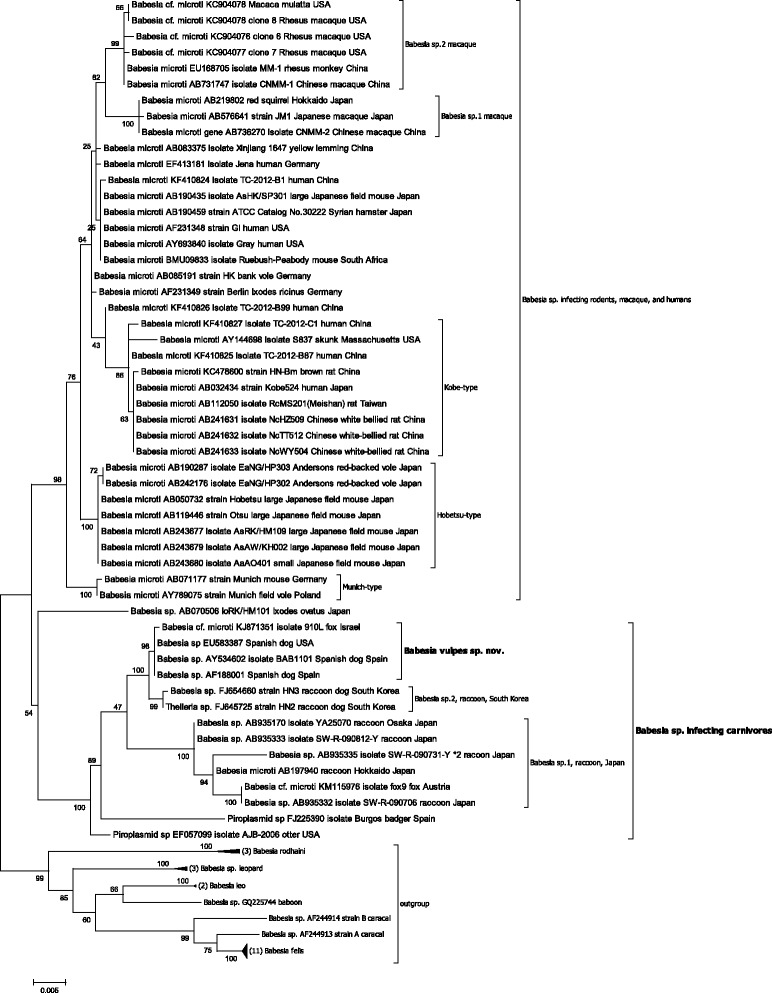
**Phylogenetic tree of near-complete** ***18S rRNA*** **gene sequences of** ***T. annae*** **,** ***B. microti*** **, and** ***B. microti*** **-related parasites using maximum likelihood.** The sequence of each isolate is labeled with its gene accession number, isolate designation, host, and geographic origin of isolation. The bootstrap values based on 1,000 replicates are displayed next to the branches. The tree is rooted using closely related *Babesia* parasites infecting rodents and carnivores of Clade I as defined in Schnittger et al. [3]. Wherever applicable, the number of pooled sequences is given. Accession number of pooled sequences are *Babesia rodhaini:* M87565, DQ641423, AB049999; *Babesia* sp. leopard [50]: JQ861967, JQ861965, JQ861972; *Babesia leo*: AY452708, AF244911; *Babesia felis*: AY452698, AY452699, AY452700, AY452700, AY452701, AY452702, AY452703, AY452704, AY452705, AY452706, AY452707. *Babesia* sp. baboon: GQ225744 [51] and *Babesia* sp. caracal AF244913, AF244914 [52]. Clades marked by brackets display a highly significant bootstrap value (≥85). The evolutionary distance is shown in the units of the number of base substitutions per site.

Figure 1 shows a phylogenetic tree based on all available near-complete sequences of the *18S rRNA* gene of *T. annae* and its synonyms, *B. microti*-related parasites, and all other related piroplasmid species of Clade I as defined in Schnittger et al. [3]. The tree branches into a group of *Babesia* sp. infecting rodents, macaque, and humans (often referred to as *B. microti*) and *Babesia* spp. that infect carnivores (often referred to as *B. microti*-related). Within the latter group, *T. annae* and related sequences derived from canines in Asia (Israel), Europe (Spain), and the USA, segregate with a highly significant bootstrap value into a single clade that defines the species *Babesia vulpes* sp. nov.

**Figure 2:**
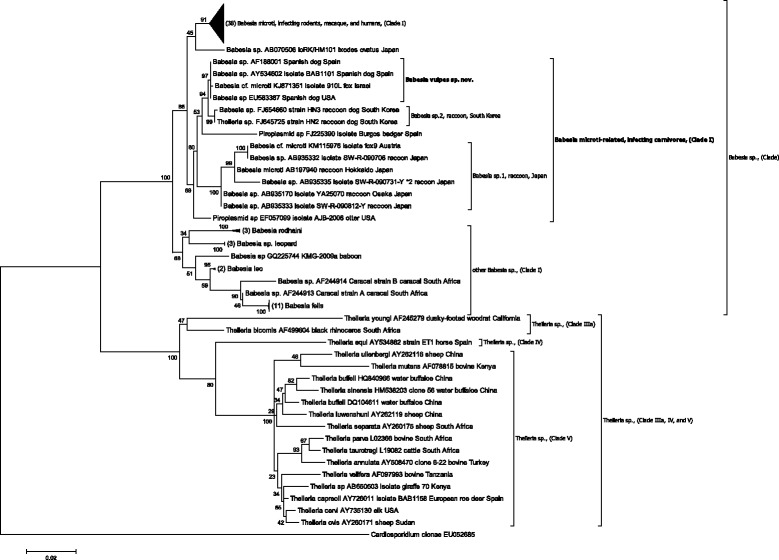
**Phylogenetic tree of near-complete** ***18S rRNA*** **gene sequences of** ***T. annae*** **,** ***Theileria*** **sp.,** ***B. microti*** **, and** ***B. microti*** **-related parasites using maximum likelihood.** The sequence of each isolate is labeled with its gene accession number, isolate designation, host, and geographic origin of isolation. The bootstrap values based on 1,000 replicates are displayed next to the branches. The tree is rooted using *Cardiosporidium cionae* as outgroup [3]. Wherever applicable, the number of pooled sequences is given. Accession number of pooled sequences are *Babesia rodhaini:* M87565, DQ641423, AB049999; *Babesia* sp. leopard [50]: JQ861967, JQ861965, JQ861972; *Babesia leo*: AY452708, AF244911; *Babesia felis*: AY452698, AY452699, AY452700, AY452700, AY452701, AY452702, AY452703, AY452704, AY452705, AY452706, AY452707. *Babesia* sp. baboon: GQ225744 [51] and *Babesia* sp. caracal AF244913, AF244914 [52]. Clades marked by brackets display a highly significant bootstrap value (≥85). The clade of *Babesia* sp. infecting rodents, macaque, and human has been collapsed. The evolutionary distance is shown in the units of the number of base substitutions per site.

In order to explicitly demonstrate different placement of *T. annae* and *Theileria* sp., a phylogenetic tree was generated following the procedure above yet including 17 relevant *Theileria* sp. sequences that belong to Clade V (*Theileria* sensu strictu), Clade IV (*Theileria equi*), and Clade IIIa (*Theileria youngi* and *Theileria bicornis*) as defined in Schnittger et al. [3] (Figure 2). Importantly, all *Theileria* sequences were found to segregate with a highly significant bootstrap into a different clade from *Babesia* sequences of Clade I, which contained also *Babesia vulpes* sp. nov.

To test the affiliation of additional incomplete *T. annae*-sequences present in the database with the above defined *B. vulves* sp. nov. clade, an additional phylogenetic analysis was carried out (Additional file 2: Figure S1). To this end, *18S rRNA* gene sequences of the same samples were used to construct the tree, but in addition other available *T. annae* and *T. annae*-related incomplete gene sequences were included. Importantly, a similar tree topology was observed as in the tree presented in Figure 1. Although, the clade of *T. annae* and *T. annae*-related sequences and its sister clade of *Babesia* sp. 2 raccoon isolates could not be distinguished by significant bootstraps, all incomplete and near-complete *T. annae* and *T. annae*-related sequences joined again into a single clade strongly suggesting their species identity.

A phylogenetic tree of all available near-complete *tubulin-beta* gene sequences of piroplasmids including a single available *T. annae* sequence is displayed in Additional file 3: Figure S2. The tree demonstrates that the *T. annae* sequence is distinct from those of *Theileria* parasites as it clusters significantly separately and distantly from *Theileria* species such as *Theileria orientalis* and *Theileria parva.* Furthermore, *T. annae* does not cluster with *B. microti* infecting rodents and humans but clusters with a *Babesia* species infecting skunks in the clade of *Babesia* infecting carnivores.

## Discussion

The goal of this study was to harmonize the phylogenetic placement of *T. annae* with its taxonomic nomenclature. In order to reevaluate the classification of *T. annae* into the genus *Theileria*, it is important to analyze the context that led to this notion in the original study describing this parasite. Zahler et al. [2] based their study assumption on a phylogenetic analysis consisting of *18S rRNA* gene sequences of some *Babesia* parasites belonging to the *Babesia* sensu stricto group, of *B. microti* and *Babesia rodhaini* from the *Babesia* sensu lato group, and of *Theileria equi*, which was thought to be a *Theileria* parasite at that time [4,33-35]. However, it has only recently been demonstrated that *T. equi* belongs to an additional distinct group within the piroplasmid order (corresponding to Clade IV in Schnittger et al. [3]), a finding that has been subsequently confirmed based on the *T. equi* genome sequence [36]. Thus, Zahler et al. [2] did not include a sequence of a relevant *Theileria* parasite (corresponding to Clade V in Schnittger et al. [3]) in their analysis. Therefore, the *18S rRNA* gene sequence of *T. annae* segregated as a relative of *B. microti* into a clade with *T. equi*, letting them assume that the parasite is related to the genus *Theileria*. Since then, in a considerable number of subsequent studies on *Babesia* and *Theileria* phylogeny, sequences of relevant *Theileria* parasites have been included, and it has been clearly demonstrated that *T. annae* sequences do not segregate with those of *Theileria* parasites but they are rather closely related to those of *B. microti* [3,26,37-42]. These studies, as well as our results, demonstrate that *T. annae* is not a *Theileria* parasite (defined as Clade V in Schnittger et al. [3]) but a parasite most closely related to *B. microti* [40] (Figure 2).

In an early pioneering study, Goethert and Telford [43] evaluated piroplasms from a range of carnivores and rodents and demonstrated that *B. microti* is an entity that seems to comprise different species. Extending their work, a number of investigators have evaluated additional piroplasmids and corroborated the existence of at least five different species lineages in this group as based on molecular phylogeny, gene structure analysis, and tick-transmission studies [3,44-46]. In accordance with its tree placement that distinguishes it from zoonotic *B. microti* parasites, *T. annae* has never been implicated as cause of human infection.

The phylogenetic tree of near-complete *18S rRNA* sequences from our study depicted in Figure 1 shows that *T. annae* segregates to form an independent and distinct clade. In several studies, relatively short amplicon fragments of the *18S rRNA* gene have been sequenced to identify *T. annae* parasites and deposited in GenBank (Additional file 1: Table S1). In order to verify their tree placement, an additional tree was constructed based on a correspondingly shorter alignment (Additional file 2: Figure S1). This tree virtually confirms the findings shown in Figure 1 and also displays a clade that comprises all *18S rRNA* genes of *T. annae* and synonyms demonstrating the species identity of these isolates*.* However, the *T. annae* clade cannot be significantly differentiated from that of *Babesia* sp. 2 raccoon, South Korean isolates, highlighting the importance of sequencing the complete *18S rRNA* gene to enhance the phylogenetic signal, and thus ensure the differentiation into clades supported by a significant bootstrap in this group of parasites.

Most phylogenetic studies on *T. annae* were performed with the *18S rRNA* gene, but the *tubulin-beta* gene likewise supports the evidence that *T. annae* is not related to *Theileria* parasites (Additional file 3: Figure S2). Furthermore, the *tubulin-beta* tree demonstrates that *T. annae* does not belong to *B. microti* which infects rodents and humans but to a separate group of *Babesia* infecting carnivores. In addition to the genetic evidence, no description has been made of a pre-erythrocytic leukocyte stage of *T. annae*, which is considered a prerequisite for inclusion in the genus *Theileria* [33,34].

Thus, phylogenetic analysis clearly shows that *T. annae* and its synonyms are a single and distinct species as demonstrated by the high genetic identity of *18S rRNA* genes from isolates originating from widespread geographic regions (Figure 1, Additional file 2: Figure S1) [2-6,10-16,18-20,47-49]. Since the red fox has been considered as the natural host/reservoir of *T. annae,* we propose to name this parasite *B. vulpes* sp. nov., after the red fox species name *V. vulpes*.

Interestingly, piroplasmids isolated from raccoons from South Korea (*Babesia* sp. 1 raccoon, South Korea) represent a sister clade of *B. vulpes* sp. nov. Other *Babesia* isolates identified in wild raccoons from Japan clearly represent a different species (*Babesia* sp. 1 raccoon, Japan) from those that have been identified in wild raccoons from South Korea (*Babesia* sp. 2 raccoon, South Korea), suggesting that these species might have a mutually exclusive endemicity. Noteworthy, a *Babesia* sp. from a fox in Austria segregates with *Babesia* sp. 2 raccoon from South Korea, suggesting that there may be an additional *Babesia* species infecting foxes besides *B. vulpes* sp. nov.

## Conclusions

This phylogenetic analysis confirms that *T. annae* does not belong to the genus *Theileria* and that it can be clearly distinguished from *B. microti* infecting rodents, macaques, and humans*.* These findings correspond with known biological characteristics as a pre-erythrocytic stage has not been demonstrated for *T. annae* and the parasite has been exclusively found to infect canines, namely foxes and dogs. Therefore, we conclude that it is a separate distinct species and propose it to be named *B. vulpes* sp. nov. The renaming of *T. annae* as *B. vulpes* sp. nov. should replace the use of synonyms like *B. microti*-like, *Babesia* cf. *microti*, *B. annae*, and *Babesia* Spanish dog isolate, prevent the current confusion to facilitate coherent scientific communication, and distinguish this parasite clearly from *Theileria* by giving it its deserved species status. In accordance with section 8.5 of the ICZN’s International Code of Zoological Nomenclature, details of the new species have been submitted to ZooBank with the life science identifier (LSID) zoobank.org:act:42884D09-A8A4-4679-BA5B-7643269C5FBF.

## Additional files

Additional file 1: Table S1. Sequences of *Theileria annae* (synonyms *Babesia* cf. *microti* and *Babesia microti*-like) amplified from foxes and deposited in the GenBank database and/or published in peer-reviewed journals. The table contains information gathered to the author’s best ability. Additional file 2: Figure S1. Phylogenetic tree of near-complete *18S rRNA* and incomplete gene sequences of *T. annae*, *B. microti*, and *B. microti*-related parasites including incomplete sequences isolated from parasites infecting dogs and foxes using maximum likelihood. The sequence of each isolate is labeled with its gene accession number, isolate designation, host, and geographic origin of isolation. The bootstrap values based on 1,000 replicates are displayed next to the branches. The tree is rooted using closely related *Babesia* parasites infecting rodents and carnivores of Clade I as defined in Schnittger et al. [3]. Wherever applicable, the number of pooled sequences is given. Accession number of pooled sequences are *Babesia rodhaini:* M87565, DQ641423, AB049999; *Babesia* sp. leopard [50]: JQ861967, JQ861965, JQ861972; *Babesia leo* [52]: AY452708, AF244911; *Babesia felis*: AY452698, AY452699, AY452700, AY452700, AY452701, AY452702, AY452703, AY452704, AY452705, AY452706, AY452707. *Babesia* sp. baboon: GQ225744 [51] and *Babesia* sp. Caracal AF244913, AF244914 [52]. Clades marked by brackets display a highly significant bootstrap value (≥85). The evolutionary distance is shown in the units of the number of base substitutions per site. The analysis involved 92 nucleotide sequences from which all positions with less than 80% site coverage were eliminated resulting in a total of 1281 positions in the final dataset. After estimation of the shape parameter [31], the T92 + G + I model was applied to infer the tree using the MEGA6 software [32]. Additional file 3: Figure S2. Phylogenetic tree of *tubulin beta* gene sequences of *T. annae*, *B. microti* and *B. microti*-related parasites using maximum likelihood. The sequence of each isolate is labeled with its gene accession number, isolate designation, host, and geographic origin of isolation. The bootstrap values based on 1,000 replicates are displayed next to the branches. The tree is rooted using *Theileria* and *Babesia* parasites of Clade V and VI as defined in Schnittger et al. [3], respectively. Clades marked by brackets display a highly significant bootstrap value (≥85). The evolutionary distance is shown in the units of the number of base substitutions per site. The analysis involved 32 nucleotide sequences of which all positions containing gaps and missing data were eliminated resulting in a total of 879 positions in the final dataset. After estimation of the shape parameter [34], the T92 + G + I model was applied to infer the tree using the MEGA6 software [35].
