# Supplementary material for: Reclassification of Theileria annae as Babesia vulpes sp. nov
Source: Parasit Vectors. 2015 Apr 8;8:207. doi: 10.1186/s13071-015-0830-5 (PMC4393874; doi:10.1186/s13071-015-0830-5)
Supplement: Supplementary file 1 [file 13071_2015_830_MOESM2_ESM.docx]

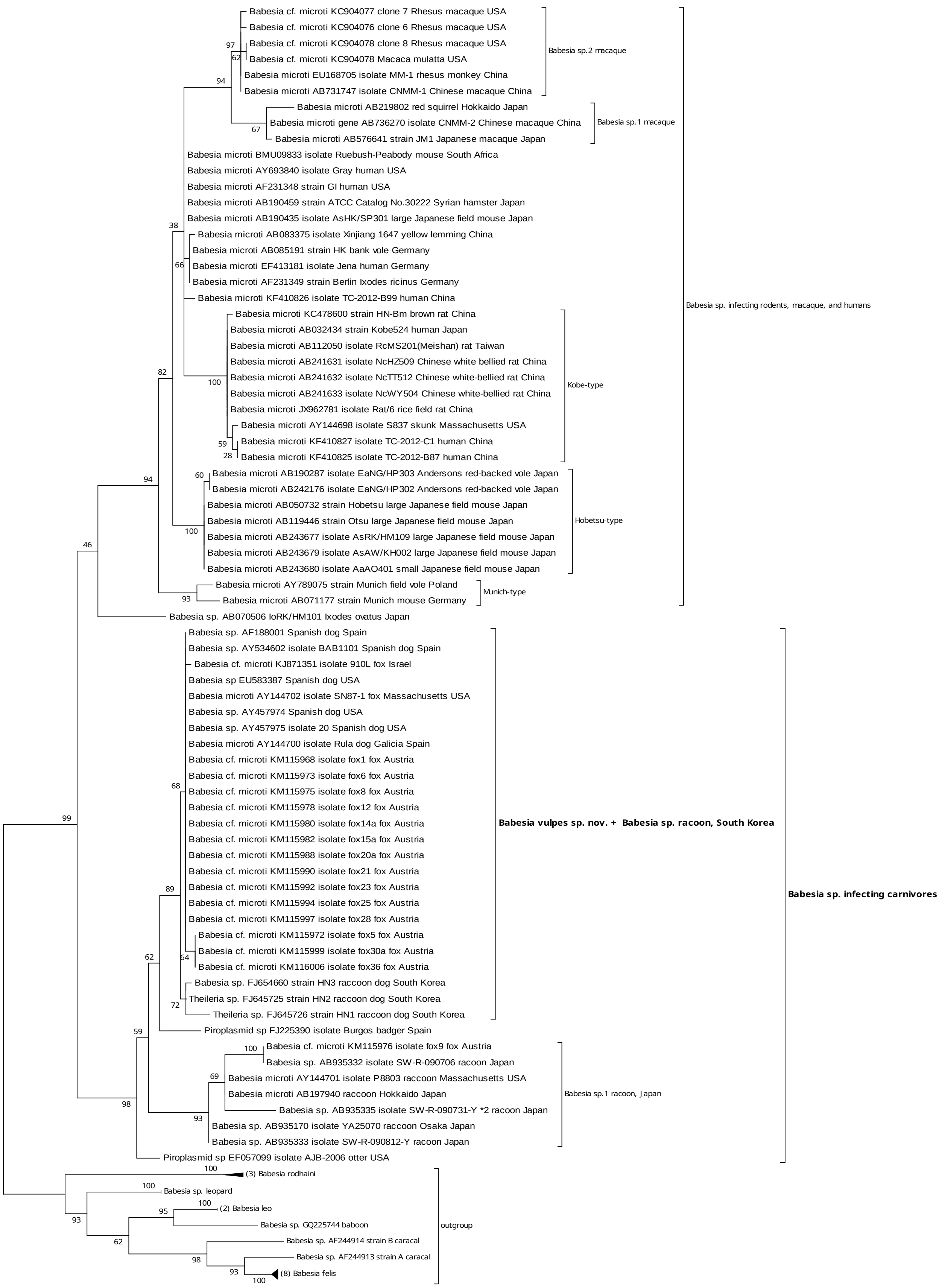
Babesia cf. microti KC904077 clone 7 Rhesus macaque USA
Babesia cf. microti KC904076 clone 6 Rhesus macaque USA
Babesia cf. microti KC904078 clone 8 Rhesus macaque USA
Babesia cf. microti KC904078 Macaca mulatta USA
Babesia microti EU168705 isolate MM-1 rhesus monkey China
Babesia microti AB731747 isolate CNMM-1 Chinese macaque China
Babesia sp.2 macaque
Babesia microti AB219802 red squirrel Hokkaido Japan
Babesia microti gene AB736270 isolate CNMM-2 Chinese macaque China
Babesia microti AB576641 strain JM1 Japanese macaque Japan
Babesia sp.1 macaque
Babesia microti BMU09833 isolate Ruebush-Peabody mouse South Africa
Babesia microti AY693840 isolate Gray human USA
Babesia microti AF231348 strain GI human USA
Babesia microti AB190459 strain ATCC Catalog No.30222 Syrian hamster Japan
Babesia microti AB190435 isolate AsHK/SP301 large Japanese field mouse Japan
Babesia microti AB083375 isolate Xinjiang 1647 yellow lemming China
Babesia microti AB085191 strain HK bank vole Germany
Babesia microti EF413181 isolate Jena human Germany
Babesia microti AF231349 strain Berlin Ixodes ricinus Germany
Babesia microti KF410826 isolate TC-2012-B99 human China
Babesia sp. infecting rodents, macaque, and humans
Babesia microti KC478600 strain HN-Bm brown rat China
Babesia microti AB032434 strain Kobe524 human Japan
Babesia microti AB112050 isolate RcMS201(Meishan) rat Taiwan
Babesia microti AB241631 isolate NcHZ509 Chinese white bellied rat China
Babesia microti AB241632 isolate NcTT512 Chinese white-bellied rat China
Babesia microti AB241633 isolate NcWY504 Chinese white-bellied rat China
Babesia microti JX962781 isolate Rat/6 rice field rat China
Babesia microti AY144698 isolate S837 skunk Massachusetts USA
Babesia microti KF410827 isolate TC-2012-C1 human China
Babesia microti KF410825 isolate TC-2012-B87 human China
Kobe-type
Babesia microti AB190287 isolate EaNG/HP303 Andersons red-backed vole Japan
Babesia microti AB242176 isolate EaNG/HP302 Andersons red-backed vole Japan
Babesia microti AB050732 strain Hobetsu large Japanese field mouse Japan
Babesia microti AB119446 strain Otsu large Japanese field mouse Japan
Babesia microti AB243677 isolate AsRK/HM109 large Japanese field mouse Japan
Babesia microti AB243679 isolate AsAW/KH002 large Japanese field mouse Japan
Babesia microti AB243680 isolate AaAO401 small Japanese field mouse Japan
Hobetsu-type
Babesia microti AY789075 strain Munich field vole Poland
Babesia microti AB071177 strain Munich mouse Germany
Munich-type
Babesia sp. AB070506 IoRK/HM101 Ixodes ovatus Japan
Babesia sp. AF188001 Spanish dog Spain
Babesia sp. AY534602 isolate BAB1101 Spanish dog Spain
Babesia cf. microti KJ871351 isolate 910L fox Israel
Babesia sp EU583387 Spanish dog USA
Babesia microti AY144702 isolate SN87-1 fox Massachusetts USA
Babesia sp. AY457974 Spanish dog USA
Babesia sp. AY457975 isolate 20 Spanish dog USA
Babesia microti AY144700 isolate Rula dog Galicia Spain
Babesia cf. microti KM115968 isolate fox1 fox Austria
Babesia cf. microti KM115973 isolate fox6 fox Austria
Babesia cf. microti KM115975 isolate fox8 fox Austria
Babesia cf. microti KM115978 isolate fox12 fox Austria
Babesia cf. microti KM115980 isolate fox14a fox Austria
Babesia cf. microti KM115982 isolate fox15a fox Austria
Babesia cf. microti KM115988 isolate fox20a fox Austria
Babesia cf. microti KM115990 isolate fox21 fox Austria
Babesia cf. microti KM115992 isolate fox23 fox Austria
Babesia cf. microti KM115994 isolate fox25 fox Austria
Babesia cf. microti KM115997 isolate fox28 fox Austria
Babesia cf. microti KM115972 isolate fox5 fox Austria
Babesia cf. microti KM115999 isolate fox30a fox Austria
Babesia cf. microti KM116006 isolate fox36 fox Austria
Babesia sp. FJ654660 strain HN3 raccoon dog South Korea
Theileria sp. FJ645725 strain HN2 raccoon dog South Korea
Theileria sp. FJ645726 strain HN1 raccoon dog South Korea
Babesia vulpes sp. nov. + Babesia sp. racoon, South Korea
Piroplasmid sp FJ225390 isolate Burgos badger Spain
Babesia cf. microti KM115976 isolate fox9 fox Austria
Babesia sp. AB935332 isolate SW-R-090706 racoon Japan
Babesia microti AY144701 isolate P8803 raccoon Massachusetts USA
Babesia microti AB197940 raccoon Hokkaido Japan
Babesia sp. AB935335 isolate SW-R-090731-Y *2 racoon Japan
Babesia sp. AB935170 isolate YA25070 raccoon Osaka Japan
Babesia sp. AB935333 isolate SW-R-090812-Y racoon Japan
Babesia sp.1 racoon, Japan
Babesia sp. infecting carnivores
Piroplasmid sp EF057099 isolate AJB-2006 otter USA
(3) Babesia rodhaini
Babesia sp. leopard
(2) Babesia leo
Babesia sp. GQ225744 baboon
Babesia sp. AF244914 strain B caracal
Babesia sp. AF244913 strain A caracal
(8) Babesia felis
outgroup
