# Supplementary material for: Reclassification of Theileria annae as Babesia vulpes sp. nov
Source: Parasit Vectors. 2015 Apr 8;8:207. doi: 10.1186/s13071-015-0830-5 (PMC4393874; doi:10.1186/s13071-015-0830-5)
Supplement: Supplementary file 2 [file 13071_2015_830_MOESM3_ESM.docx]

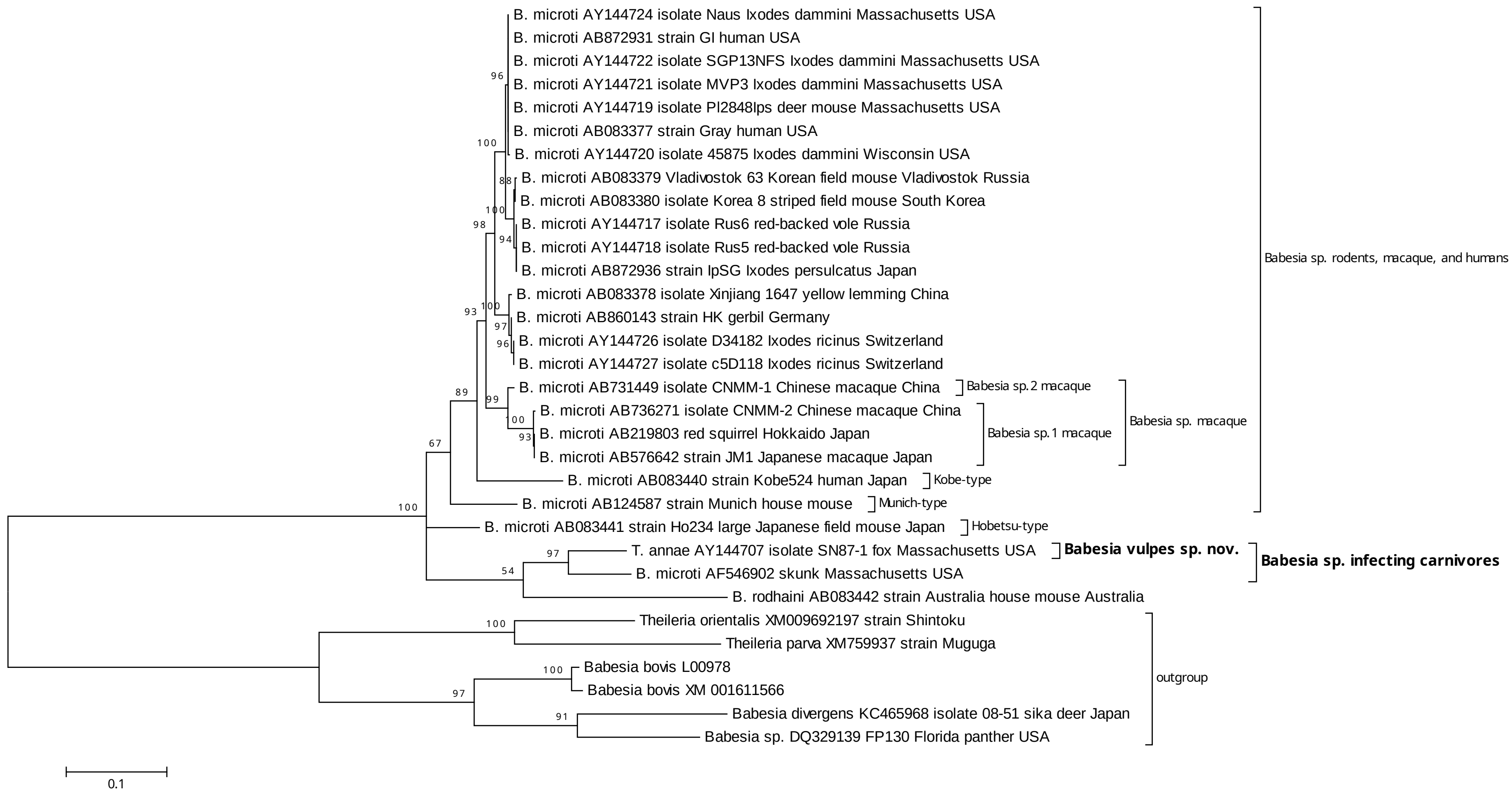
B. microti AY144724 isolate Naus Ixodes dammini Massachusetts USA
B. microti AB872931 strain GI human USA
B. microti AY144722 isolate SGP13NFS Ixodes dammini Massachusetts USA
B. microti AY144721 isolate MVP3 Ixodes dammini Massachusetts USA
B. microti AY144719 isolate PI2848Ips deer mouse Massachusetts USA
B. microti AB083377 strain Gray human USA
B. microti AY144720 isolate 45875 Ixodes dammini Wisconsin USA
B. microti AB083379 Vladivostok 63 Korean field mouse Vladivostok Russia
B. microti AB083380 isolate Korea 8 striped field mouse South Korea
B. microti AY144717 isolate Rus6 red-backed vole Russia
B. microti AY144718 isolate Rus5 red-backed vole Russia
B. microti AB872936 strain IpSG Ixodes persulcatus Japan
B. microti AB083378 isolate Xinjiang 1647 yellow lemming China
B. microti AB860143 strain HK gerbil Germany
B. microti AY144726 isolate D34182 Ixodes ricinus Switzerland
B. microti AY144727 isolate c5D118 Ixodes ricinus Switzerland
B. microti AB731449 isolate CNMM-1 Chinese macaque China
B. microti AB736271 isolate CNMM-2 Chinese macaque China
B. microti AB219803 red squirrel Hokkaido Japan
B. microti AB576642 strain JM1 Japanese macaque Japan
B. microti AB083440 strain Kobe524 human Japan
B. microti AB124587 strain Munich house mouse
B. microti AB083441 strain Ho234 large Japanese field mouse Japan
T. annae AY144707 isolate SN87-1 fox Massachusetts USA
B. microti AF546902 skunk Massachusetts USA
B. rodhaini AB083442 strain Australia house mouse Australia
Theileria orientalis XM009692197 strain Shintoku
Theileria parva XM759937 strain Muguga
Babesia bovis L00978
Babesia bovis XM 001611566
Babesia divergens KC465968 isolate 08-51 sika deer Japan
Babesia sp. DQ329139 FP130 Florida panther USA
Babesia sp. rodents, macaque, and humans
Babesia sp.2 macaque
Babesia sp.1 macaque
Babesia sp. macaque
Kobe-type
Munich-type
Hobetsu-type
Babesia vulpes sp. nov.
Babesia sp. infecting carnivores
outgroup
96
100
88
100
98
94
93
100
97
96
89
99
100
93
67
100
97
54
100
100
97
91
0.1
