# Supplementary material for: Reclassification of Theileria annae as Babesia vulpes sp. nov
Source: Parasit Vectors. 2015 Apr 8;8:207. doi: 10.1186/s13071-015-0830-5 (PMC4393874; doi:10.1186/s13071-015-0830-5)
Supplement: Additional file 1: Table S1. — Sequences of Theileria annae (synonyms Babesia cf. microti and Babesia microti-like) amplified from foxes and deposited in the GenBank database and/or published in peer-reviewed journals. The table contains information gathered to the author’s best ability. [file 13071_2015_830_MOESM1_ESM.docx]

**Additional file 1: Table S1. Sequences of *Theileria annae* (synonyms *Babesia* cf. *microti* and *Babesia microti*-like) amplified from foxes and deposited in the GenBank database and/or published in peer-reviewed journals.** The table contains information gathered to the author’s best ability.

| **Year of fox sampling** | **Geographic region** | **Fox species** | **Gene** | **GenBank accession numbers** | **Reference** |
| --- | --- | --- | --- | --- | --- |
| 1997-1999 | Central Spain | *Vulpes vulpes*^a^ | 18S rRNA | AY150069 | [11] |
| 1997-1999 | Central Spain | *Vulpes vulpes* | 18S rRNA | No new accession; 100% identical to AY150069 | [47] |
| 2004-2006 | Northern and central Italy | *Vulpes vulpes* | 18S rRNA | BO365-SE | [12] |
| 2006-2007 | Northern Spain | *Vulpes vulpes* | 18S rRNA | No new accession; 100% identical to AY150069 | [48] |
| 2006 | Canada (Prince Edward Island) | *Vulpes vulpes* | beta-tubulin | No new accession;100% identical to AY144702 and AY144709 | [14] |
| ND | Canada (Prince Edward Island) and USA (North Carolina) | *Vulpes vulpes* | 18S rRNA | No new accession;≥99.9% identical to AY144700 and AY144702 | [15] |
| ND | USA (North Carolina) | *Urocyon cinereoargenteus*^b^ | 18S rRNA | No new accession;≥99.9% identical to AY144700 and AY144702 | [15] |
| ND | Canada (Prince Edward Island) and USA (North Carolina) | *Vulpes vulpes* | beta-tubulin | No new accession;≥98.8% identical to AY144707 | [15] |
| ND | USA (North Carolina) | *Urocyon cinereoargenteus* | beta-tubulin | No new accession;≥98.8% identical to AY144707 | [15] |
| 2007-2008 | Croatia (west and north) | *Vulpes vulpes* | 18S rRNA | HM212628 | [13] |
| ND | Poland (northeast) | *Vulpes vulpes* | 18S rRNA | FJ480420 | [29] |
| 2008-2010 | Portugal | *Vulpes vulpes* | 18S rRNA | JN793479 (21 sequence consensus) | [16] |
| 2008-2012 | Italy (Alps) | *Vulpes vulpes* | 18S rRNA | KF773740 and KF773741 | [19] |
| 2009 | Germany (Thuringia) | *Vulpes vulpes* | 18S rRNA | JX679165 | [17] |
| 2009 | Germany (Thuringia) | *Vulpes vulpes* | 18S rRNA | 3 sequences: JX679166 to JX679168 | Najm et al. (unpublished) |
| 2011 | Hungary | *Vulpes vulpes* | 18S rRNA | 14 sequences: KM232509 to KM232522 | [20] |
| 2013-2014 | Bosnia and Herzegovina | *Vulpes vulpes* | 18S rRNA | No new accession; 99-100% identical to HM212628 and AY457974 | [49] |
| 2014 | Austria (Lower Austria – northeast) | *Vulpes vulpes* | 18S rRNA | 18 sequences: KM115968, KM115972, KM115973, KM115975, KM115976, KM115977, KM115978, KM115980, KM115982, KM115985, KM115988, KM115990,KM115992, KM115994, KM115997, KM115999, KM116004, KM116006 | [18] |
| 2014 | Israel | *Vulpes vulpes* | 18S rRNA | 6 sequences: KJ871347 to KJ871352 | Margalit Levi et al. (unpublished) |

^a^Red fox; ^b^Gray fox; ND: not defined.
